# Phased repatriation of Lebanese expatriates stranded abroad during coronavirus disease 2019 (COVID-19) pandemic

**DOI:** 10.1186/s13690-021-00740-y

**Published:** 2021-11-23

**Authors:** Dalal Youssef, Atika Berry, Nada Ghosn, Mahmoud Zalzali, Riad Fadlallah, Linda Abou-Abbas, Hamad Hassan

**Affiliations:** 1 Preventive Medicine Department, Ministry of Public Health, Beirut, Lebanon; 2 Epidemiological Surveillance Program, Ministry of public Health, Beirut, Lebanon; 3 Ministry of Public Health, Beirut, Lebanon

**Keywords:** Repatriation, Coronavirus disease (COVID-19), Pandemic, Lebanon

## Abstract

**Background:**

The coronavirus disease (COVID-19) pandemic represents a serious worldwide threat. Stranded Lebanese citizens abroad appealed to the Lebanese government to embark on citizen repatriation missions. We aim to document the Lebanese experience in the repatriation of citizens during COVID-19 which allow us to disclose encountered challenges and lessons learned.

**Methods:**

This is a retrospective description of processes involved in the phased repatriation of Lebanese citizens. The Mission consisted of 4 phases starting, April 5th until June 19th 2020. The prioritization of returnees was based on both medical and social risk assessment. The repatriation team was divided into four groups: the aircraft team, the airport team, the hotel team and the follow up team. On arrival, all returning citizens were tested using Polymerase chain Reaction (PCR) based technique, and were obliged to adhere to a mandatory facility quarantine for 24 to 48 h. Returning travelers who were tested positive for COVID-19 were transferred to the hospital. Those who were tested negative were urged to strictly comply with home-quarantine for a duration of 14 days. They were followed up on a daily basis by the repatriation team.

**Results:**

Overall, 25,783 Lebanese citizens have returned home during the phased repatriation. The third phase ranked the uppermost in regard of the number of citizens repatriated. The total number of performed PCR tests at the airport upon arrival was 14,893 with an average percentage of around 1% positivity for COVID-19. On the other hand, more than 10,687 repatriates underwent external PCR requisite in the third and fourth phases. Two hundred seventy-two repatriates were tested positive for COVID-19 upon their arrival.

**Conclusion:**

Considering the limited human and financial resources besides the economic and political crisis, the overall repatriation mission could be considered as a successful experience. Such processes would not have been achieved without the professionalism of all involved stakeholders.

## Background

The coronavirus (COVID-19) outbreak represents a serious threat to public health. Firstly identified in Wuhan, China towards the end of 2019, it is sprouting into the largest worldwide health and mobility crisis ever seen [1, 2]. Following its consideration as public health emergency of international concern on 30th of January 2020, the COVID-19 was declared by the World Health Organization (WHO) as a pandemic on 11th of March 2020 [3]. As the numbers of cases rise, the pandemic continues to hinder human movement, with knock-on effects for immigration and border management regimes [4].

Under the requirements of International Health Regulations (IHR) 2005, it is mandatory that health authorities are prepared to contain COVID-19 by inaugurating effective contingency plans and measures at international ports, airports and ground crossings. Additionally, it is required to establish and improve an active surveillance system that is able to early detect cases and culminated by isolating and management of cases as well as tracing their contacts [5]. In this regard, many countries have imposed entry bans or quarantines for citizens or recent travelers to the affected countries [6, 7]. Despite that lockdowns and other appropriate mobility restrictive measures are crucial to save lives, these procedures may also sternly impact negatively the economies and can delay the transport of critical goods and services. Hence, warnings and concerns have been raised over the efficiency of travel restrictions to control the spread of COVID-19 [8].

Considering the threat of introduction of COVID-19 and its consequential negative impact on the general population, as well as the potential to giving ride to community transmission, triggering complications and death in high-risk groups, Lebanon has been vigilant and proactive in responding to Covid-19 since January 2020. In this regard, series of measures have been rolled out starting with issuing guidance for travelers visiting affected areas accompanied by an advice to avoid non-essential journeys to COVID-19 hotspots [9, 10]. A National Committee for COVID-19 (NCC) was established, focusing on leading and running the COVID-19 national preparedness and response [11]. In addition, as part of the preparedness plan, and since the early stage of the pandemic, a range of preventive measures were put in place at points of entry, prior to the identification of the first imported COVID-19 case. Screening of travelers coming from infected areas at points of entries was performed since the initial phase accompanied by dissemination of health messages and travel notices apprising travelers on common symptoms of COVID-19 and how to seek medical service if needed. Moreover, a form collecting contact information of the traveler, exposure and symptoms was filled by the traveler upon arrival. Demographic and contact information were transmitted to the ministry of public health (MOPH) for rapid evaluation and further follow-up. These preventive measures have played a crucial role in the detection of the first documented case of COVID-19 infection coming from Iran to Lebanon on February 21st, 2020. In the light of the political and economic crisis that affected the country deeply, which has already dared with limited health system resources, the Lebanese population was about to encounter another opponent revealed by the upsurge of COVID-19 imported cases [12]. Thus, the government took harsh containment measures including the closure of the airport. This step was subsequent to some previous measures consisting on restriction flights from a number of COVID-19 affected countries [13]. A ban on flights to and from Iran, Italy, China and South Korea was announced, while Lebanese citizens and their families in other countries were given 4 days to return before flights would also be ceased to and from those countries.

In March 14, 2020, with the upsurge of COVID-19 cases to 99, Lebanon declared a state of health emergency and imposed a two-week lockdown as part of the country’s efforts to slow the spread of the virus. The stated measures included closure of Beirut airport, as well as sea and land ports, from March 18 until March 29, except for diplomatic missions and cargo aircraft. Hence, Lebanese residents were urged to stay home except for urgent trips, but no curfew was declared. On other hand, many countries started the closure of their borders for international and local travel. Such measures urged stranded Lebanese citizens abroad to appeal to Lebanese government to embark on citizen repatriation missions. However, akin process is challenging for many countries, since it may lead to spikes of COVID-19 cases after periods of containment [11]. As per expatriates’ plea, the Lebanese Cabinet approved on March 31st plans for special flights to bring home expatriates trapped abroad by the COVID-19 outbreak. Thus, an exceptional opening of the airport for Middle East Airlines (MEA) flights took place in order to organize the phased repatriation process.

The mission objectives were to safely repatriate as many citizens based on aircraft’s capacity without threatening their safety and to prevent alongside transmission of the disease to the Lebanese population. As part of a pandemic preparedness and response plan, it is of great interest to document the Lebanese experience in the repatriation of citizens during COVID-19 in addition to identify existing gaps and unveil encountered challenges. Also, the following document highlights some of the lessons learned from this experience of taking a proactive rights-based approach which can draw point to promote more effective interventions and to better enlighten future implementation and decision-making.

## Methods

This is a retrospective description of processes involved in repatriation of Lebanese citizens from various countries during the Covid-19 pandemic. The Mission was led by the NCC in coordination with the MOPH.

### Preparation

As per Lebanese expatriates appeal, Lebanese government developed a phased repatriation plan, while keeping safety and wellbeing at the forefront of this assignment. In addition, more elements were considered, such as medical history of the person and any accompanying family members. Length and ability to travel safely and further strains on elements of hardship for the repatriate (e.g., access to quality medical facilities, an underlying threat of political unrest, limited goods and services) were taken into consideration as well.

First of all, the Lebanese Ministry of Foreign Affairs initiated the coordination with embassies abroad to find out how many individuals want to return to Lebanon at their own expense. Then, each Lebanese citizen, living abroad and wishing to come back to Lebanon was requested to fill an identification form. It contained demographic and clinical information about the person in addition to a specialized part focusing on the availability and the suitability of appropriate quarantine location in Lebanon.

Before embarkation: Exit screening was conducted before departure for early detection of symptoms and corresponding results were shared with health authorities in Lebanon. Suspected cases, detected through exit screening are requested accordingly to delay their travel and to undergo further evaluation and treatment.

To accomplish this mission, the MOPH team was divided into four groups: the aircraft team, the airport team, the hotel team and the follow up team.

Aircraft team: The flight Team personnel consisted of two medical personnel, one physician and one health officer. Considering the limited number of human resources currently available at MOPH, volunteers have actively participated in the composition of this team All flight Teams trained personnel underwent briefing on in-fight safety procedures and use of personal protective equipment (PPE) and how to deal with suspected case of COVID-19 on board. Such procedures were pivotal to reduce exposure and limit transmission to other passengers or to aircraft crew. The non-medical crew of the aircraft were also appropriately instructed and outfitted, as well as conscious of the signs and symptoms that could appear on symptomatic COVID-19 cases. Upon boarding the flight, wearing face masks was compulsory for passengers as well as hands sanitization. At the entrance of the aircraft, the medical personnel performed visual triaging to identify those who appeared unwell or required special assistance. Seating was already arranged and mapped by MEA company based on the feasibility and the flight capacity in order to provide safe distance between repatriates. Such mapping was crucial for duly dealing with any passenger starting to show symptoms and requiring isolation and also for the identification of those in the immediate vicinity. The passengers were also divided into three categories (red, green and yellow) according to their health status. Passengers were asked to minimize their movement and to avoid eating during flight. Light meals were only offered for flight duration of more than 5 h. All of these factors were crucial towards control of spread of COVID-19 during flight [14, 15].

#### Airport team

The airport Team personnel consisted of physicians, health officers and laboratory technicians. The team performed another triage using temperature screening and visual checking to detect and segregate symptomatic passengers. Hence, any passenger presenting symptoms was transported directly by the Red Cross to the hospital for testing and further evaluation. Given their health status revealed by the color of the worn wristband, the passenger debarkation process occurred in several groups starting by people with underlying health conditions followed by the healthy and asymptomatic passengers and lastly by the aircraft team. At the airport, repatriates undertook several processes including decontamination, health screening, swab screening and boarding of the bus that will ferry them to a designated hotel. Furthermore, unbreakable personal items were also sanitized. Screening swab test consisted of collecting nasopharyngeal swabs from each returning citizen to be tested using Polymerase Chain Reaction (PCR) based technique except for newborns. The Flight Team underwent similar process as the repatriates. The airport team verified that each passenger has filled the identification form which included contact information and dedicated quarantine location details. Passengers were requested to sign a consent that obliged them to adhere to the requisite quarantine. In addition, health messages and brochures informing repatriates on Covid-19 symptoms, quarantine requirements and how to seek medical support in case of symptoms onset, were distributed to the passengers. The MOPH also shared with them the dedicated hotline of the COVID-19 in case they needed any further information. Asymptomatic passengers were transported by an assigned bus to the designated hotels.

#### Hotel team

The Lebanese Government has assigned hotels to host returnees for 24 to 48 h, which corresponds to the duration needed until the release of laboratory results for travelers. At the hotel, there was a MOPH team, called hotel team that coordinated with the hotel staff to ensure both safety of travelers and staff. Each traveler was requested to stay in his room and any further movement between rooms was forbidden. Based on the laboratory results, repatriates who tested negative were allowed to return to their home to finalize the mandatory quarantine for 14 days. No special transport means have been assigned for the repatriates who tested negatives for COVID-19. The later were transferred from the hotel to their home by non-emergency vehicles such as taxi service or by private transport (relative/acquaintances)” to complete quarantine up to 14 days. In case of availability of a private vehicle, it was preferable that the repatriate drives himself; however, this choice was not applicable in most cases. To minimize the risk of contamination during transportation, the ministry of Public Health has issued strict instructions for safe transportation of repatriates. These instructions required from the personal (driver, relative …) who is involved in transporting of a repatriate to apply infection prevention and control practices such as wearing a facemask and ensuring adequate ventilation, including opening vehicle’ windows during the transportation. Repatriates were also required to wear face masks. Children younger than 2 or anyone who has difficulty breathing were exempted from these requirements and were asked to practice social distancing from the driver as much as possible. In addition, commonly touched surfaces in the vehicle should be cleaned and disinfected at the beginning and end of each shift. However, the positive passengers were notified by the MOPH team who coordinated with the Red Cross as well to arrange their transfer to the hospital. It is noteworthy that all positive passengers, either showing symptoms or not, were transferred to Rafic Hariri University hospital to undergo a very clear medical assessment in order to ensure that their health status is stable enough and didn’t require a special attention. Positive cases with unstable health status and those having a condition hindering their ability to self-isolate were hospitalized, whereas asymptomatic cases and those with stable health status were discharged for home-based isolation.

#### Follow up team

The follow up team contacted the repatriates on a daily basis, whether present at home or facility quarantine, during the 14 days following their arrival to Lebanon, in order to monitor their symptoms. Besides, they were asked about their physical and psychological needs. Revealed physical needs, including appropriate accommodation and supplies were referred to the local authorities to explore with them the possibility to provide any support based on their capacities. Those starting to show symptoms were referred to the hospital for re-testing.

In addition to their role in monitoring symptoms among repatriates, the follow up team played a crucial role in raising awareness regarding measures and procedures to be followed during quarantine period and to provide emotional support in order to evade panic. They encouraged them to seek appropriate care when developing symptoms as well. Lastly, the follow up team reminded them regularly about the COVID-19 hotline in case they need any information.

### The repatriation process was executed in 4 phases

#### Phase 1

The time frame of the first phase was from 5th to 12th April 2020. MEA operated successive repatriation trips to evacuate Lebanese expatriates and nationals from abroad. It is mandatory to fill out the required medical form as a condition for boarding and send it back to MOPH.

#### Phase 2

Lebanon has suspended repatriation flights until April 27th in order to maintain the capacity of hospitals and hotels designated for isolation and to re-evaluate the implemented measures. The second phase of Lebanese repatriation started on April 28th, and extended until May 8th 2020. Newly detected COVID-19 cases in Lebanon included repatriated, and some returning expats who have flouted the requirement of quarantine, raising fears that the returnees could threaten progress made in controlling the virus. Fines and legal actions were taken to deter violators.

#### Phase 3

The phase 3 scheduled flights from the 14th of May till the 24th of May 2020 inclusive. The Phase 3 repatriation flights were subject to the following conditions: PCR test should be done 3 days before the departure in laboratories contracted with the Lebanese Embassy or in any laboratory recognized by the country of departure. In addition, the test result should not exceed 72 h. However, PCR test cannot be replaced by a doctor’s report.

#### Phase 4

The fourth phase of Lebanese repatriation flights runs from 11th to 19th June 2020. Eighteen flights returning Lebanese nationals stranded abroad have been scheduled. Passengers departing from countries that can administer PCR tests were seated side by side on aircrafts.

## Results

**Fig. 1:**
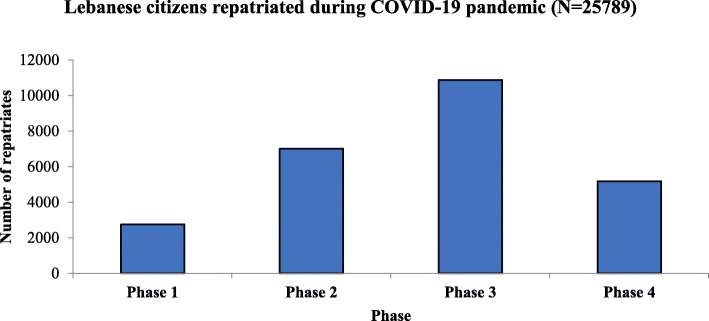
Lebanese citizens repatriated during COVID-19 pandemic (*N* = 25,783)

**Fig. 2:**
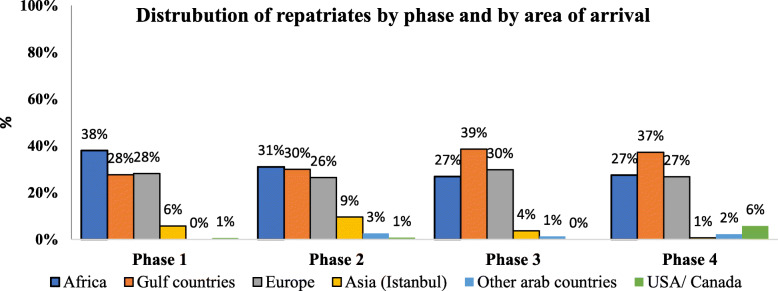
Distribution of repatriates per phase and per area of arrival

**Fig. 3:**
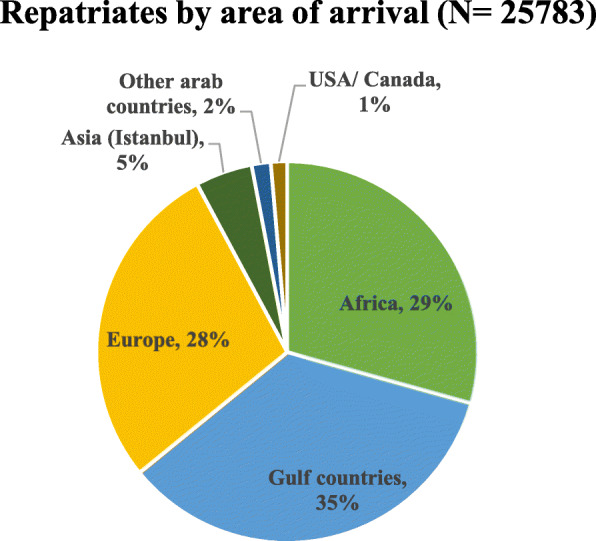
Distribution of repatriates per area of arrival

The repatriation mission consisted of 4 phases with a period extending between April 5th and June 19th, 2020. Overall, 25,783 Lebanese citizens have been returned home during the phased repatriation. Figure 1 displayed the number of Lebanese citizens returned to Lebanon in each phase either transported by commercial or by private jets. More than 1200 repatriates were transferred to Lebanon through private jets and the remaining (24507) were repatriated by commercial flights led by MEA. The third phase ranked the uppermost of the phases in regard of the number of citizens repatriated (10,866 returnees). Figure 2 and Fig. 3 showed the distribution of repatriates per area of arrival. The majority of repatriates were returning from Africa, Europe and Gulf Countries.

Table 1 summarizes laboratory tests results of the returnees during each phase of the repatriation. A notable increase in the number of returnees was noted in the second and the third phases of repatriations where nearly 69.2% of repatriates returned in these 2 phases. For the phase 1 and 2, external PCR test was not requested from passengers; hence all returnees were tested except newborns and military staff and those who had certificate that they have already performed a negative PCR test outside. Of note, military staff was not tested at their arrival due to security reasons. However, they applied very strict precautionary measures and followed their own standard protocols including testing, quarantine and isolation which are in line with WHO and MOPH guidelines. Additionally, they were tested at country of departure. Some PCR tests performed abroad were repeated at the airport. The overall number of performed PCR testing at airport during the phased repatriation on arrival was 13,013 tests with an average percentage of test positivity around 1%. On the other hand, more than 12,245 repatriates underwent external PCR required in the third and fourth phase before their arrival to Lebanon. All repatriates traveling to Lebanon during the latest two phases were requested by the MOPH to fill out an online health declaration before departure in which they declared if they have already performed the PCR test. Those who have performed a lab test were asked to attach a scan of the negative PCR test. The other ones were selected for PCR testing upon arrival. Of note, some laboratory results issued from some countries were not considered reliable due to limited laboratory capacities in these countries or due to the applied self-sampling methods. In this case, repatriates were selected for PCR testing upon arrival. Test screening showed that 272 repatriates were tested positive for COVID-19 upon their arrival. The highest positive percentage of internally performed test was recorded among citizen who returned during the third phase of repatriation whether in commercial flights (3.42%) or private jets (3.45%). However, this issue didn’t affect the percentage of positive cases recorded among repatriates during the same phase (1%). To be noted, 510 PCR tests from the repatriates were turned positive subsequently during the quarantine period.

**Table 1:** Summary of the repatriates per Laboratory testing

Repatriation phases	Repatriates	External PCR	Internal PCR (airport)	Laboratory results (PCR at airport)			
				Positive	Negative	% positive among repatriates	% positive among internally tested repatriates
	n	n	n	n	n	%	%
Phase 1	2742	195	2225	32	2193	1.17%	1.43%
Phase 2	7000	1363	5637	75	5598	1.07%	1.33%
Phase 3	10,866	6814	3994	104	3890	0.96%	2.6%
Phase 4	5175	3873	1157	61	1096	1.18%	5.27%
Total all phases	25,783	12,245	13,013	272	12,777	1.05%	1.8%

The overall percentage of positive COVID-19 cases for the overall repatriation mission was 1.05% which correspond to 272 cases. It was approximately the same during the different phases of the process. Figure 3 showed the percentage of positive cases recorded between repatriates during the repatriation process. The majority of positive cases (61%) were recorded between repatriates returning from Africa followed by those returning from Gulf Countries (19%) and Europe (15%). Table 2 summarizes the distribution of positive cases by phase and by area of repatriation.

**Table 2:** Distribution of positive cases per phase and per area of arrival

Positive cases per area of arrival										
	Phase 1		Phase 2		Phase 3		Phase 4		Total positive	
	n	%	n	%	n	%	n	%	n	%
Africa	2	6%	46	61%	77	74%	41	67%	166	61%
Gulf countries	1	3%	12	16%	25	24%	13	22%	51	19%
Europe	27	84%	6	8%	2	2%	7	11%	42	15%
Asia (Istanbul)	2	6%	11	15%	0	0%	0	0%	13	5%
Other arab countries	0	0%	0	0%	0	0%	0	0%	0	0%
USA/ Canada	0	0%	0	0%	0	0%	0	0%	0	0%
Total	32	100%	75	100%	104	100%	61	100%	272	100%

## Discussion

The COVID-19 pandemic has highlighted the duty of repatriation that countries owe for citizens well-being and how this, sometimes, requires extreme measures [16]. Lebanese citizens around the world have faced huge challenges as many of them were left stranded, unable to return home. There was massive appeal for repatriation causing dilemma given the infectiousness of COVID-19 and the risk of importation. Lebanon started the first phase of repatriating expats on April 5th amid apprehensions. Of the estimated 20,000 Lebanese nationals who registered to return from abroad, more than 25,000 have been repatriated in the phased repartition process. Prior to the repatriation of Lebanese citizens, the epidemiological situation of COVID-19 in Lebanon was considered under control and no community transmission was recorded so far. Out of the total, 272 positive cases were detected at the airport. Since several repatriates were carelessness and didn’t comply with the quarantine rules, hence these positive cases were the primary drivers of clusters in the community, leading later to an upsurge of cases.

Thus, an increase trend in the number of COVID-19 cases was clearly observed. Furthermore, these positive cases were the primary drivers of clusters before full border reopening.

Screening at airport, quarantine and monitoring of repatriates are considered as an effective tool for preventing and slowing importation of infectious disease into a country [17]. However, screening alone is considered an imperfect barrier to spread due to: the absence of detectable symptoms during the incubation period; variation in the severity and detectability of symptoms once the disease begins to progress; or active evasion of screening by travelers [18]. In addition, it necessitates precise and correct contact information for travelers in order to be operative. However, incorrect and incomplete traveler contact information provided by some repatriates led to wasting substantial time to address this issue as well as compromising timely contact of them or completely impeding the reach out of some repatriates. This could be due to the manual method of repatriate data collection, in addition to the fact that some repatriates have intentionally provided biased contact information. Considering this fact, a switch to an electronic method of data collection was implemented during the latest phases of the repatriation, it thereby facilitated timely case identification, follow-up and contact investigations. Electronic messaging platforms, such as text messaging, were used to inform repatriates about their laboratory results. In addition, an application was developed by the MOPH, called “daily follow up”, to encounter the limited human resources. Consistent with previous international disease outbreaks, screening and quarantine of travelers were most functioning when disease-ridden passengers could be readily recognized and when tracking of cases using available public health resources [19]. Combining visual and test screening at the airport allow us to capture infected but asymptomatic repatriates on arrival [20]. However, monitoring the repatriates during the period of follow-up was a labor-intensive for MOPH team given the huge number of repatriates and the frequency of daily flights.

Based on post flights debriefing reports, which identified aspects of team performance and suitability of the adopted processes, planning and work procedures along the repatriation mission were updated [21]. Indeed, this tip had assisted us to better organizing future flights and for determining opportunities for improvement at the individual, team, and mission level. Among the improvements that were made, requesting external PCR from repatriates in the third and fourth in addition to progress made of data collection methods.

Despite that the public health experts have previously cautioned against repatriation which could rapidly overwhelm the country’s health care system unless strict quarantining and monitoring procedures are implemented [22]. Despite this, not all repatriates have adhered to the strict quarantine. Indeed, in a country already beset by economic and financial crises, dearth in COVID-19 facility able to provide mandatory quarantine for repatriates limit the capacity of accurate on-site monitoring and preclude the closely follow-up of repatriates. In addition, lack of awareness among community hampered the effectiveness of social distancing measures. Some returned citizens turned a blind eye to the required self-quarantine and spread the infection onto others through their carelessness. This comes on the heels of over 40 confirmed coronavirus infections, which have been directly linked to a woman who arrived from Saudi Arabia. The woman had failed to adhere to self-quarantine measures and infected family and friends. In addition, some repatriated individuals, who should have been in self-quarantine were reported to have been indulging in large social gatherings. This highlights the meagerness of enforcement mechanisms and punitive measures to ensure serious implementation and compliance with measures. On the other hand, some of the municipalities failed in playing a vital role in term of follow up and monitoring of COVID-19 cases and contact. This is could be due to the fact that most of the municipalities lack the resources required to respond adequately in enforcing quarantine and providing needed items to repatriates. In addition, data was not always shared timely with municipalities, hence causing delays in identification of quarantined cases. However, allowing municipalities to adequately participate can enhance the respect of the quarantine and isolation measures. Additionally, their insights regarding the situation of repatriates within their authority zones should also be integrated in the decision-making process given that they are at the forefront whenever crises strike at locality level. Limited coordination among other sectors beyond health was noticed.

During the repatriation, it was never more important than that population, particularly repatriates understand the situation and what exactly is expected of them [23]. Good communication was the cornerstone of this as it can cut the noise of misinformation [24]. Considering this fact, the MOPH was proactive and transparent in displaying results and reliable information to the public. Furthermore, the MOPH team played a crucial role in raising awareness and reducing panic among repatriates through phone calls and diffused internal clear and concise messages.

In the regard of laboratory testing of repatriates, performing external PCR testing was not requested during the first two phases. False-negative PCR tests were reported between external PCR, this can be due to inaccuracy of tests in some countries. In fact, there are likely various reasons for the false-negative results it can be due to sampling errors like incorrectly swabbing. Timing of swabbing is also a major factor as the virus moves around the body throughout the infection [25]. Given that the PCR test has a high specificity yet moderate sensitivity; a positive test hold more weight than a negative one.

Concerning the fees of tickets, the expatriates have paid for their own pockets, but critics have complained about steep fares amidst a domestic financial crisis and severe restrictions on transactions from Lebanese bank accounts [26]. Furthermore, some reports flooded various media channels with claims that the decision around who was allowed to return was overwhelmed by networks. It is noteworthy that few returnees didn’t have suitable accommodation in Lebanon to move into during quarantine period. As such, they may need to move into temporary accommodation,, during their time back in the home location. This led to questions regarding who should meet such costs taking into account the economic crisis in Lebanon.

Some key lessons were identified through this mission to guide similar future intervention that should operate during moments of crisis.

Firstly, in the preparedness and response to unpredictable crisis such as COVID-19, it is crucial to keep contingency plans up-to-date as these can guide management operations as well as highlighting mitigating actions. Additionally, the country needs to be goal orientated and to consider risk, based on the available assets. Close collaboration and coordination between different stakeholders as well as transparency and timely sharing of information were the cornerstone of a successful response. Flexibility and well-defined role and responsibilities are also considered a crucial trait during this hectic situation. Bearing in mind that internal and external communication were keys as often local organizations and countries need to collaborate with each other to mitigate the fallout caused by a crisis. External communication was particularly important for our repatriation mission, as often the evacuations required communication with different international governments and points of entries.

It’s clear that no one emergency is the same and there is never going to be a perfect playbook guiding us what to do at every stage. Based on clear principles which inform good practice, evacuations may happen at the last minute but successful strategies are months or years in the planning.

## Conclusion

Considering the limited human and financial resources besides the economic crisis and the politic concern, the overall repatriation mission could be considered as a successful experience despite the presence of some gaps in enforcement and coordination. Such processes would not have been achieved without the professionalism and hard work of all involved stakeholders.

## Data Availability

The datasets used and analyzed during the current report will not be made publically available but will be available from the corresponding author on reasonable request.

## Abbreviations

COVID-19: Coronavirus Disease 2019
MEA: Middle East Airlines
MOPH: Ministry of Public Health
IHR: International Health Regulations
WHO: World Health Organization
NCC: National Committee for COVID-19
PCR: Polymerase Chain Reaction
